# Mortality risk and causes of death in exogenous Cushing's syndrome: a systematic review and meta-analysis

**DOI:** 10.1210/clinem/dgag123

**Published:** 2026-03-24

**Authors:** Padiporn Limumpornpetch, Paul M Stewart, Mar Pujades-Rodriguez, Paul D Baxter, Ana Tiganescu, Victoria Nyawira Nyaga, Ann W Morgan

**Affiliations:** Faculty of Medicine and Health, University of Leeds, Leeds, LS2 9NL, UK; Endocrinology and Metabolism Unit, Division of Internal Medicine, Faculty of Medicine, Prince of Songkla University, Hat Yai, Songkhla 90110, Thailand; Faculty of Medicine and Health, University of Leeds, Leeds, LS2 9NL, UK; NIHR Leeds Biomedical Research Centre, Leeds Teaching Hospitals NHS Trust, Leeds, LS1 3EX, UK; Faculty of Medicine and Health, University of Leeds, Leeds, LS2 9NL, UK; Leeds Institute of Cardiovascular and Metabolic Medicine, University of Leeds, Leeds, LS2 9JT, UK; Leeds Institute of Cardiovascular and Metabolic Medicine, University of Leeds, Leeds, LS2 9JT, UK; Unit of Cancer Epidemiology, Belgian Cancer Centre, Sciensano, Brussels 1050, Belgium; Faculty of Medicine and Health, University of Leeds, Leeds, LS2 9NL, UK; NIHR Leeds Biomedical Research Centre, Leeds Teaching Hospitals NHS Trust, Leeds, LS1 3EX, UK; NIHR Leeds Medtech and in-Vitro Diagnostics Co-Operative, Leeds Teaching Hospitals NHS Trust, Leeds, LS1 3EX, UK

**Keywords:** exogenous Cushing's syndrome, mortality, glucocorticoids, systematic review, meta-analysis

## Abstract

**Context:**

Chronic oral glucocorticoids (GCs) are widely prescribed for multiple diseases. Although long-term GC exposure causes systemic toxicity, the magnitude, dose-response relationship, and causes of GC-associated mortality remain incompletely defined.

**Objective:**

To quantify overall and cause-specific mortality associated with chronic oral GC exposure and evaluate dose-response relationships across underlying disease groups.

**Methods:**

We conducted a systematic review and meta-analysis following PRISMA guidelines (PROSPERO CRD42017067530). PubMed/MEDLINE, EMBASE, Cochrane Library, Web of Science, and CINAHL were searched from 1945 to March 2019. Eligible studies reported standardized mortality ratios (SMRs) or absolute deaths among adults receiving chronic oral GCs. Random-effects meta-analysis and mixed-effects meta-regression were used to synthesize mortality and dose-response associations across GC exposure metrics, exposure duration, and disease subgroups.

**Results:**

One-hundred and sixteen studies encompassing 128 cohorts and 51 380 patients were included. Chronic GC exposure was associated with excess mortality (pooled SMR 1.87; 95%CI 1.32–2.61; I^2^ 74.0%). The pooled proportion of death was 12.0% (95%CI 10–14%). Mortality risk was highest in inflammatory diseases (30.0%) and the vasculitides (18.0%). Higher cumulative dose (≥5 g prednisolone-equivalent), average daily dose >5 mg/day, and higher initial dose were each independently associated with increased mortality. Cardiovascular disease was the leading causes of death (25.6%), followed by malignancy (15.7%) and infection (13.4%).

**Conclusion:**

Chronic oral GC exposure was associated with increased mortality across disease groups, with higher cumulative and early-treatment doses correlating with higher risk. However, causal attribution remains uncertain due to confounding by indication, limited disease-severity data, and exposure misclassification. These findings support GC stewardship and targeted cardiovascular and infection risk mitigation, rather than indiscriminate dose reduction.

Glucocorticoids (GCs) remain indispensable therapeutic agents for autoimmune, inflammatory, hematologic, and malignant diseases across global clinical practice (1). With 0.7% to 3.4% of adults identified as chronic oral GC users in recent population-based studies (2-7), the systemic toxicity associated with long-term GC use, often termed exogenous Cushing's syndrome (CS), constitutes an urgent clinical and public-health concern. Chronic GC exposure leads to recognized side effects, including metabolic, cardiovascular, infectious, neuropsychiatric, and thromboembolic complications (1, 8-11) that are comparable to, but not identical with, endogenous CS. However, despite decades of clinical application, the precise mortality burden directly attributable to chronic oral GC therapy has remained insufficiently characterized. Most published studies have focused primarily on the underlying disease being treated, rather than the direct effects of GC dose and treatment duration on mortality. Furthermore, the evidence base for exogenous CS-related mortality has been constrained by study design variability, substantial confounding factors, inconsistent reporting of GC exposures, and different risk attribution models (12). To address these critical limitations and provide clear, quantifiable estimates, we conducted the first comprehensive systematic review and meta-analysis specifically focused on chronic oral GC exposure, analyzing both the magnitude and specific causes of death.

## Methods

The study adhered to the Preferred Reporting Items for Systematic Reviews and Meta-analysis (PRISMA) guideline (12, 13) and was prospectively registered in PROSPERO (CRD42017067530; Supplemental 1) (14). All protocol steps were undertaken by PL and MPR and independently verified by AWM and PMS; any discrepancies were resolved through structured consensus meetings involving all authors.

### Search strategy

A comprehensive search of PubMed/MEDLINE, Cochrane Library, EMBASE, Web of Science, and CINAHL was conducted for studies published from 1945 to March 2019 (Supplemental 2) (14). Search strategies included prespecified, population- and outcome-focused search terms for CS, including any Cushing* or types of oral GCs, and terms for the study outcome (death and mortality) were used. Reference lists of eligible studies or prior systematic reviews were hand-searched to identify additional articles. When essential outcome or exposure data were unavailable, corresponding authors were contacted directly to request missing information.

### Selection criteria

Eligible studies were English-language original articles reporting standardized mortality ratios (SMRs) or absolute number of deaths among adults (≥18 years) receiving chronic oral GCs. Chronic oral GC use was stringently defined as >5 mg/day of prednisolone-equivalent for >3 weeks, with GCs comprising at least 90.0% of exposure among participants (15). To minimize small-study effects, a minimum sample size of 50 participants was required (16). Studies were excluded if they met any of the following: (1) nonhuman studies; (2) case reports, case series, conference abstracts, book chapters, systematic reviews, or clinical guidelines; (3) cohorts with intrinsically high background mortality unrelated to GC such as malignancy; transplant recipients, severe infections including human immunodeficiency virus (HIV), malaria, sepsis, tuberculosis, viral hepatitis, or intensive care populations; (4) alcohol- or liver-related diseases (cirrhosis, alcoholic hepatitis, or autoimmune hepatitis); (5) nonoral GC routes or studies involving replacement therapy; (6) non-English language; or (7) endogenous CS. To avoid duplications, when multiple publications used overlapping datasets, the most comprehensive dataset (largest sample size and longest follow-up) was selected a priori.

### Outcome definitions

The primary outcomes were SMR, comparing observed deaths to expected deaths in age-, sex-, and nationality-matched reference populations, and the overall cause mortality proportion at the longest follow-up. Secondary outcomes included cause-specific mortality.

### Review procedures and data extraction

Duplicate records were identified and removed using Endnote X9 and Rayyan [4]. Potential overlapping cohorts were systematically identified by cross-referencing study setting, population, and observation period. Title/abstract and full-text screening was performed in Covidence (17). Data extraction was performed into standardized Microsoft® ACCESS (Office® 365) forms and included PubMed identifiers, first author, country, hospital and publication year, study design, demographics, care setting, recruitment period, data source, GC indication, and all reported GC dose metrics (cumulative, average, maintenance, initial, and last follow-up dose). Exposure duration and follow-up times were extracted as distinct variables to optimize characterization of cumulative risk. For studies reporting multiple eligible cohorts (eg, systemic lupus erythematosus populations from different countries), data were extracted separately provided each cohort contained ≥50 participants. Mortality measures (SMR, number of deaths, percentage, and causes of death) were fully extracted. All extracted data underwent independent cross-checked by PL, MPR, PMS, and AWM to ensure accuracy and resolve discrepancies.

### Quality assessment

Risk of bias was assessed independently by 2 investigators (PL and MPR) using a modified ROBINS-I tool for non-randomized studies of interventions (18). Disagreements were resolved through discussion and consensus.

Seven domains were evaluated in accordance with ROBINS-I guidance: (1) confounding, (2) selection bias, (3) classification and diagnosis of GC exposure, (4) deviations from intended interventions, (5) missing data, (6) measurement of mortality outcomes, and (7) reporting bias (Supplemental 3) (14). Prespecified confounding domains included underlying disease severity and inflammatory burden, comorbidities, duration of disease and follow-up, GC exposure, and concomitant therapies. Each domain-level and overall study quality was classified as low, moderate, serious, critical, and uncertain (18). Overall study-level risk was defined as the highest level of bias attributed to any individual domains. Studies categorized as having a serious risk of bias were considered to have important limitations in at least one domain (most commonly confounding or selection bias) that could influence the magnitude of the observed association but did not preclude inclusion in the quantitative synthesis. Studies categorized as critical risk of bias were considered to have fundamental methodological limitations that precluded reliable interpretation of the results and were therefore excluded from quantitative synthesis (Supplemental 3, Tables S9 and S10) (14).

### Data synthesis, meta***-***analysis, meta***-***regression, and subgroup analysis

SMRs represented the number of exogenous CS deaths in the study compared to the expected number of deaths in an age- sex- and nationality-matched control population. SMRs were pooled using a DerSimonian–Laird random-effects model with inverse-variance weighting of log-SMRs; summary estimates were generated by exponentiation of log-SMR. The proportion of deaths was synthesized using mixed-effects logistic meta-regression under a binomial distribution model, yielding logit-transformed pooled mortality estimates. Between-study heterogeneity was quantified using *I*^2^, interpreted as: <25% low, 25% to 49% moderate, 50% to 74% substantial, and ≥75% high heterogeneity (19). Prespecified meta-regression analyses examined sources of heterogeneity across GC dose metrics (cumulative, mean, maintenance, initial, and last follow-up), treatment duration, underlying disease indications, and follow-up length. Disease-specific subgroups were analyzed using mixed-effects logistic models incorporating relevant GC exposure variables. Cause-specific mortality outcomes were narratively summarized where reported. All analyses were conducted using STATA version 16.1 (Stata Corp, Texas, USA) (20) with “*metan*” (21) and “*metapreg*” (22) packages. Sensitivity analyses excluded studies with serious, critical, or unclear risk of bias to evaluate the robustness of the primary outcomes.

### Role of the funding source

The funder was not involved in the study design, data extraction, statistical analyses, data interpretation, or manuscript preparation.

## Results

### Study selection and characteristics

**Figure 1:**
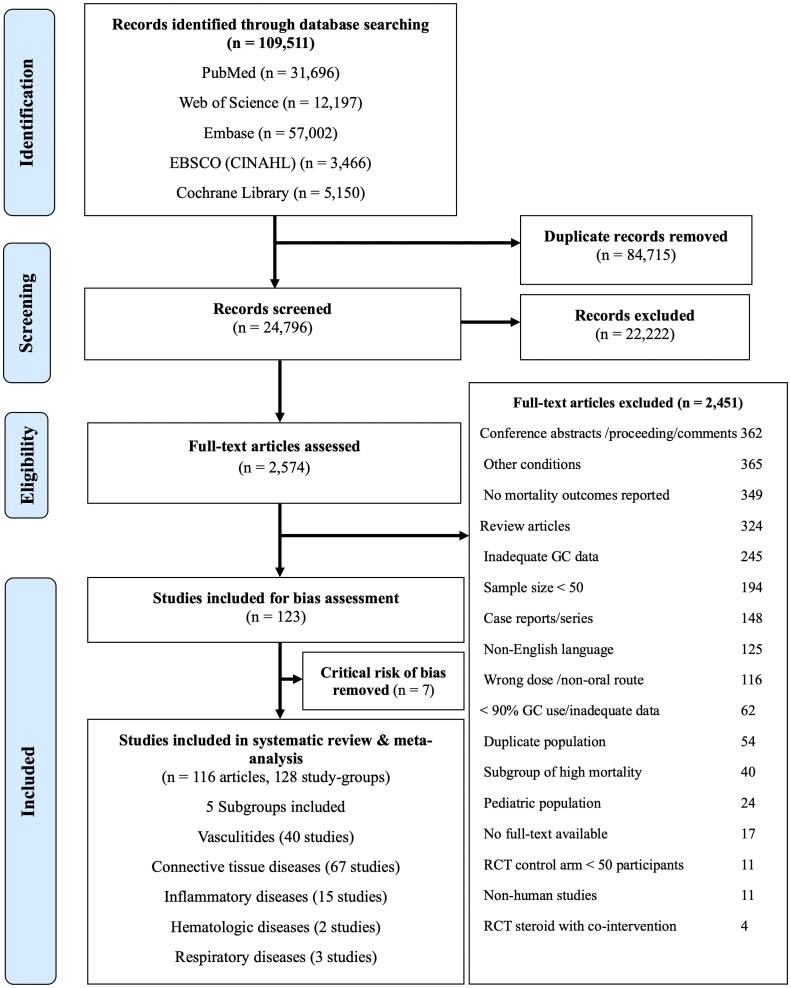
Prisma flow diagram for searching for exogenous Cushing’s Syndrome and mortality (24). Flow diagram illustrating the identification, screening, eligibility assessment, and inclusion of studies evaluating mortality associated with exogenous glucocorticoid exposure. Reasons for exclusion at the full-text stage are detailed. Abbreviations: GC, glucocorticoid; n, number of articles; N, number of patients; RCT, randomized controlled trial.

A PRISMA flow diagram (23) is presented in Fig. 1. The search identified 109 511 articles, of which 84 715 duplicates were removed. After screening titles and abstracts, 2574 full-text articles were assessed for eligibility. A total of 123 articles met initial criteria; however, 7 were excluded because of critical risk of bias (24-30). The final dataset 116 articles, representing 128 study cohorts and 51 380 adults receiving chronic oral GC (Table 1, Supplemental 4–6) (14).

**Table 1:** Characteristics of the 116 articles (128 study groups) included in the systematic review of exogenous Cushing's syndrome

1st Author (year)	Country	Study design Level of care	No. patients	Observation period	Age at diagnosis mean [Median]	Data source	women (%)	deaths (%)	Follow-up in years [Median]	GC type	Duration of GC use (months) mean [Median]
**Vasculitides: giant cell arteritis**											
Bengtsson 1981 (31)	Sweden	Retro [S]	90	NR	71.0	Med	67 (74.4)	13 (14.4)	4.9	PRN	59.0
Chevalet 2000 (32)	France	Prospective cohort [C]	164	1992–NR	73.3	Med	116 (70.7)	5 (3.0)	NR	PRL	12.0
Gran 2001 (33)	Norway	Retro [S]	64	1987–1997	NR	Med	49 (76.6)	13 (20.3)	5.3	PRL	NR
Hachulla 2001 (34)	France	Retro [S]	133	1977–1995	72.0	Registry	95 (71.4)	41 (30.8)	5.6	PRL & PRN	40.0
Uddhammar 2002 (35)	Sweden	Retro [S]	136	1973–1995	70.4	Med	NR	114 (83.8)	[10]	PRL	[35]
Les 2015 (36)-medium dose	Spain	Retro [S]	53	2004–2012	74.7	Med	37 (69.8)	1 (1.9)	[2.9]	PRN	NR
Les 2015 (36)-high dose	Spain	Retro [S]	50	2004–2012	73.3	Med	31 (62.0)	3 (6.0)	[2.9]	PRN	NR
Labarca 2016 (37)	US	Retro [S]	286	1998–2014	75.0	Med	213(74.5)	69 (24.1)	[5.1]	NR	NR
Wilson 2017 (38)	UK	Retro [C]	5011	1987–2013	72.9	Med	3713(74.1)	517 (10.3)	[1.8]	PRL	NR
Ly 2017 (39)	France	Prospective cohort [S]	428	1976–2014	75.0	Med	274 (64.0)	143 (33.4)	5.1	PRL	NR
**Vasculitides: Takayasu arteritis**											
Park 2005 (40)	Korea	Retro [S]	94	1991–2003	[29.5]	Med	NR	6 (6.4)	5.1	PRL	NR
**Vasculitides: ANCA-associated vasculitis**											
Hoffman 1992 (41)	US	Retro [S]	158	NR	[41.0]	Med	79 (50)	32 (20.3)	NR	PRN	[12]
Koldingsnes 2002 (42)	Norway	Retro [S]	56	1984–2000	[50.3]	Registry	21 (37.5)	13 (23.2)	[4.7]	PRL	[24]
Slot 2003 (43)	Netherlands	Retro [S]	85	1982–2001	56.0	Meds	30 (35.3)	37 (43.5)	5.0	PRN	NR
Harper 2005 (44)	UK	Retro [S]	229	1990–2000	[65]	Med	105 (45.9)	91 (39.7)	5.0	PRL	60.0
Rihova 2005 (45)	Prague, Czech Republic	Retro [S]	61	1986–1997	[54]	Med	24 (39.3)	19 (31.1)	NR	PRN	NR
Holle 2011 (46)	Germany	Retro [S]	155	1966–1997	[48]	Meds	79 (51)	22 (14.2)	[6.6]	PRL	NR
Holle 2011 (46)	Germany	Retro [S]	167	1999–2005	[55]	Med	82 (49.1)	8 (4.8)	[3.9]	PRL	NR
Holle 2011 (46)	Germany	Retro [S]	123	1994–2005	[52]	Med	61 (49.6)	13 (10.6)	[7.3]	PRL	NR
McGregor 2012 (47)	US	Retro [S]	61	2000–2009	[63]	Registry	23 (37.7)	8 (13.1)	2.9	PRL	20 [13]
Gregersen JW, 2012 (48)	Denmark	Retro [S]	50	1999–2007	[66]	Med	47 (94)	8 (1.06)	1.0	PRL	12.0
Specks 2013 (49)	US	Retro [S]	98	NR	[51.5]	Med	46 (46.9)	2 (2)	0.5	PRN	5.5
Specks 2013 (49)	US	Retro [S]	99	NR	54.0	Med	54 (54.5)	2 (2)	0.5	PRN	5.5
Nakaya 2013 (50)	Japan	Retro [S]	64	2000–2010	69.0	Med	25 (39.1)	22 (34.4)	3.3	PRL	6.0
Moosig 2013 (51)	Germany	Retro [S]	150	1990–2009	49.1	Med	74 (49.3)	12 (8.0)	7.7	PRL	53.0
Lai 2014 (52)	China	Retro [S]	398	1997–2011	[66]	Med	205 (51.5)	135 (33.9)	[2.2]	PRN	NR
Andreiana 2015 (53)	Romania	Retro [S]	75	2000–2014	[60]	Med	39 (52)	24 (32)	[3.2]	PRL	38.4
Yamagata 2016 (54)	Japan	Retro [S]	150	2002–2012	70.0	Med	89 (59.3)	32 (21.3)	[2.0]	PRL	NR
Fukui 2016 (55)	Japan	Retro [S]	81	2000–2015	71.0	Med	47 (58.0)	9 (11.1)	NR	PRL	NR
Haris 2017 (56)	Hungary	Retro [S]	101	1998–2013	61.4	Med	61 (60.4)	60 (59.4)	2.6	PRL	NR
Pu 2017 (57)	China	Retro [S]	123	2004–2012	61.9	Med	59 (48)	46 (37.4)	1.4	PRN	NR
Abe 2017 (58)	Japan	Retro [S]	52	2002–2014	73.2	Med	28 (53.8)	27 (51.9)	2.1	PRL	NR
Judge 2017 (59)	UK	Retro [S]	232	1990–2011	[64]	Med	86 (37.1)	74 (31.9)	[1.0]	PRN	NR
Solans-Laqué 2017 (60)	Spain	Retro [C]	450	1990–2014	[54.2]	Med	223 (49.6)	129 (28.7)	[82]	PRL	NR
Shobha 2018 (61)	India	Retro [S]	60	2002–2012	44.0	Med	25 (41.7)	11 (18.3)	4.7	PRL	NR
**Vasculitides: antiglomerular basement membrane disease**											
Huart 2016 (62)	France	Retro [C]	122	1983–2006	[31.0]	Registry	45 (36.9)	16 (13.1)	1.0	PRL	12.0
**Vasculitides: central nervous system vasculitis**											
Salvaran 2015 (63)	US	Retro [S]	159	1983–2011	[48.0]	Med	91 (57.2)	15 (9.4)	[1.0]	PRN	[9]
**Vasculitides: medium and small vessel vasculitis**											
Bourgarit 2005 (64)	France	Retro [S]	595	1953–1999	52.2	Med	243 (40.8)	145 (24.4)	6.4	PRL	NR
Mathew 2007 (65)	UK	Retro [S]	106	1976–2004	[58.7]	Med	NR	16 (15.1)	NR	PRL	NR
Alibaz-Oner 2017 (66)	US	Retro [S]	89	1980–2014	[51.1]	Med	46 (51.7)	7 (7.9)	6.5	PRN	NR
**Connective tissue diseases: systemic lupus erythematosus (SLE)**											
Wallace 1982 (67)	US	Retro [S]	230	1950–1980	27.0	Med	200 (87.0)	82 (35.7)	10.0	PRL	NR
Harisdangkul 1984 (68)	US	Retro [S]	79	1977–1981	34.1	Med	71 (89.9)	16 (20.3)	3.5	PRL	NR
Hashimoto 1992 (69)	Japan	Retro [S]	141	NR	[28.7]	Med	NR	18 (12.8)	6.0	PRN	NR
Shayakul 1995 (70)	Thailand	Retro [S]	569	1984–1993	28.0	Med	515 (90.5)	89 (15.6)	3.2	PRL	NR
Huong 1999 (71)	France	Retro [S]	180	1980–1993	27.0	Med	147 (81.7)	24 (13.3)	9.1	other	NR
Illei 2001 (72)	US	Clinical trial [S]	82	1986–1999	NR	Med	68 (82.9)	11 (13.4)	11.0	PRN	NR
Illei 2002 (73)	US	Retro [S]	145	1981–1990	[29.2]	Med	NR	5 (3.4)	[10.1]	PRN	NR
Badsha 2002 (74)	Singapore	Retro [S]	55	1989–2000	[35.2]	Med	46 (83.6)	6 (10.9)	0.5	PRL	6.0
Liang 2004 (75)	China	Retro [S]	162	1991–2001	28.8	Med	131 (80.9)	26 (16)	NR	PRN	NR
Mok 2004 (76)	Hong Kong	Retro [S]	189	1988–2001	31.1	Med	167 (88.4)	7 (3.7)	8.0	PRL	NR
Mikdashi 2004 (77) NPDI = 0	US	Retro [S]	64	1992–2003	37.0	Med	121 (93.1)	0(0)	7.0	PRN	NR
Mikdashi 2004 (77) NPDI ≥ 1	US	Retro [S]	66	1992–2003	37.0	Med	121 (93.1)	8 (12.1)	7.0	PRN	NR
Tang 2009 (78)	China	Retro [S]	94	1985–2004	27.9	Med	84 (89.4)	5 (5.3)	3.2	PRN	NR
Patel 2011 (79)	US	Prospective cohort [S]	86	NR	32.0	Med	72 (83.7)	24 (27.9)	10.0	PRN	NR
Lopez 2012 (80)	UK	Retro [S]	350	1991–NR	[36.0]	Med	322 (92)	34 (9.7)	[9]	PRN	[108]
Arends 2012 (81)	Netherlands	Observational study[S]	50	1995–2009	NR	Med	NR	5 (10)	[9.6]	PRL	NR
Ayodele 2013 (82)	South Africa	Retro [S]	66	1995–2009	[30.2]	Med	61 (92.4)	26 (39.4)	4.7	NR	NR
Moroni 2013 (83)	Italy	Retro [S]	89	1968–2012	28.8	Med	84 (94.4)	6 (6.7)	[21.9]	PRL	NR
Fatemi 2013 (84)	Iran	Retro [S]	82	1994–2010	32.3	Med	65 (79.3)	5 (6.1)	[8]	PRL	NR
Mok 2014 (72)	Hong Kong	Clinical trial [S]	74	2005–2012	36.2	Med	70 (94.6)	0 (0)	0.5	PRN	6.0
Mok 2014 (72)	Hong Kong	Clinical trial [S]	76	2005–2012	36.1	Med	68 (89.5)	1 (1.3)	0.5	PRL	6.0
Mahmoud 2015 (LN) (85)	Egypt	Retro [S]	135	1999–2011	24.4	Med	129 (95.6)	17 (12.6)	4.6	PRN	NR
Koo 2016 (86)	Korea	Retro [S]	193	1980–2008	31.2	Med	167 (86.5)	10 (5.2)	13.2	PRL	26.0
Jung 2016 (87)	South Korea	Retro [S]	230	1997–2015	[41.8]	Med	194 (84.3)	13 (5.7)	5.5	PRL	NR
Pego-Reigosa 2016 (88)	Spain	Cross-sectional study [C]	1918	2011–2012	[35.0]	Med	NR	155 (8.1)	[8.8]	PRL	NR
Joo 2017 (89)	Korea	Prospective cohort [S]	1120	1998–2012	[27.4]	Med	1031 (92.1)	53 (4.7)	NR	PRL	NR
Sheane 2017 (90)	Canada	Retro [S]	173	1970–2015	33.8	Med	147 (85)	23 (13.3)	15.1	PRL	96.0
Mahmoud 2018 (91)	Egypt	Retro [S]	770	2002–2015	[22.1]	Med	707 (91.8)	33 (4.3)	6.1	NR	NR
Wei 2011 (92) *SLE with pregnancy	China	Retro [S]	86	2005–2010	28.2	Med	86 (100)	0 (0)	[0.7]	PRN	8.7
Yang 2014 (93) *SLE with pregnancy	China	Retro [S]	82	1992–2012	NR	Med	NR	5 (6.1)	1.3	PRN	9.0
**Connective tissue diseases: Bullous disease**											
Krain 1974 (94)	US	Retro [S]	59	1955–1973	[64.5]	Med	23 (39.0)	13 (22)	5.0	PRL	36.0
Rosenberg 1976 (95)	US	Retro [S]	107	1955–1970	NR	Med	53 (49.5)	48 (44.9)	NR	PRL	3.0
Joly 2002 (96)	France	Clinical trial [S]	171	NR	[81]	Med	111 (64.9)	62 (36.3)	1.0	NR	12.0
Seo 2003 (97)	Korea	Retro [S]	51	1993–2001	46.8	Med	29 (56.9)	1 (2.0)	2.0	PRL	32.1
Shahidi-Dadras 2007 (98)	Iran	Clinical trial [S]	51	1997–2003	46.9	Med	25 (49.0)	1 (2)	1.4	PRL	12.0
Shahidi-Dadras 2007 (98)	Iran	Clinical trial [S]	72	1997–2003	42.6	Med	36 (50.0)	0 (0)	1.0	PRN	12.0
Mimouni 2010 (99)	Israel	Retro [S]	155	1976–2004	53.5	Med	94 (60.6)	16 (10.3)	NR	PRN	72.0
Kim 2011 (100)	Korea	Retro [S]	199	1993–2008	[46.1]	Med	102 (51.3)	13 (6.5)	3.9	PRL	NR
Zhang 2013 (101)	China	Retro [S]	80	2005–2010	[71.0]	Med	41 (51.3)	35 (43.8)	2.7	PRN	NR
Cai 2014 (101)	Singapore	Retro [S]	316	2004–2009	[75.7]	Med	NR	139 (44)	3.0	PRL	NR
Bai 2016 (102)	China	Retro [S]	68	2008–NR	[52.7]	Med	NR	6 (8.8)	3.1	PRL	NR
Kalinska-Bienias 2017 (103)	Poland	Retro [S]	65	2000–2013	[76.2]	Med	NR	21 (32.3)	2.3	PRN	NR
Williams 2017 (104)	UK Germany	Clinical trial [S]	121	NR	77.2	Med	57 (47.1)	25 (20.7)	[1.0]	PRL	1.5
**Connective tissue diseases: dermato/polymyositis**											
Henriksson 1982 (105)	Sweden	Retro [S]	79	1967–NR	52.2	Med	45 (57.0)	25 (31.6)	5.2	PRL	27 [19]
Agarwal 2006 (106)	US	Retro [S]	53	1991–2002	48.8	Med	38 (71.7)	7 (13.2)	5.3	PRN & other	NR
Naji 2010 (107)	Iran	Retro [S]	65	–NR	34.5	Med	44 (67.7)	3 (4.6)	NR	PRL	7.9
Uchino 2012 (108)	Japan	Retro [S]	115	1970–2009	55.5	Med	82 (71.3)	19 (16.5)	14.3	PRL	NR
Schiopu 2012 (109)	US	Retro [S]	160	1997–2003	48.4	Med	116 (72.5)	27 (16.9)	[4.6]	NR	NR
Taborda 2014 (110)	UK	Retro [S]	90	1976–2007	[38.5]	Med	64 (71.1)	13 (14.4)	[11.5]	PRN	NR
Johnson 2015 (111)	US	Retro [S]	100	1990–2011	[50.1]	Med	65 (65.0)	6 (6.0)	3.0	PRN	36.0
Galindo-Feria 2016 (112)	Mexico	Retro [S]	69	1985–2012	[46]	Med	51 (73.9)	6 (8.7)	[5.8]	PRL	NR
Galindo-Feria 2016 (112)	Mexico	Retro [S]	264	1985–2012	[40]	Med	201 (76.1)	48 (18.2)	[2.9]	PRL	NR
Nuño-Nuño 2017 (113)	Spain	Retro [S]	467	1980–2014	[41.1]	Med	348 (74.5)	113 (24.2)	[9.7]	PRL	76.9
**Connective tissue diseases: glomerulonephritis**											
Rose 1971 (114)	UK	Clinical trial [S]	56	1967–1970	[34.2]	Med	NR	6 (10.7)	0.5	PRN	6.0
**Connective tissue diseases: IgA nephropathy**											
Goumenosl 1995 (115)	UK	Retro [S]	66	NR	[40]	Med	20 (30.3)	3 (4.5)	[3.8]	PRL	24.0
**Connective tissue diseases: IgM nephropathy**											
Kuthong 2000 (116)	Thailand	Retro [S]	72	1978–1996	24.5	Med	35 (48.6)	1 (1.4)	5.0	PRL	NR
**Connective tissue diseases: nephrotic syndrome**											
Nolasco 1986 (117)	UK	Retro [S]	79	1963–1982	[42]	Med	39 (49.4)	13 (16.5)	7.6	PRL	NR
du Buf-Vereijken 2004 (118)	Netherlands	Retro [S]	56	1991–2002	50.0	Med	10 (15.4)	5 (8.9)	4.3	PRN	NR
Funabik 1992 (119)	Japan	Retro [S]	65	–NR	[42]	Med	NR	8 (12.3)	8.0	PRL	NR
Shin 2012 (120)	Korean	Retro [S]	122	1990–2009	49.5	Med	50 (41.0)	2 (1.6)	5.2	PRL	NR
**Connective tissue diseases: idiopathic pulmonary fibrosis**											
Tukiainen 1983 (121)	Finland	Prospective cohort [S]	100	1967–1979	53.0	Med	51 (51.0)	44 (44)	6.8	PRL	34.8
Park 2009 (122)	Korea	Retro [S]	83	1991–2006	[54.4]	Med	56 (67.5)	24 (28.9)	[4.4]	PRL	17.4
**Connective tissue diseases: myasthenia gravis**											
Evol 2000 (123)	Italy	Retro [S]	113	1978–1998	[68.5]	Med	NR	9 (8.0)	NR	PRN	54.6
**Connective tissue diseases: sarcoidosis**											
Johns 1974 (124)	US	Retro [S]	152	1962–1972	30.2	Med	112 (73.7)	15 (9.0)	4.0	PRN	41.6
Kandolin 2015 (125)	Finland	Retro [S]	62	2010–2014	48.6	Med	48 (77.4)	7 (11.3)	[1.4]	PRN	12.0
**Connective tissue diseases: autoimmune thrombocytopenia**											
Jacobs 1986 (126)	South Africa	Retro [S]	134	1971–1981	NR	Med	NR	2 (1.5)	3.0	PRN	NR
Portielje 2001 (127)	Netherlands	Retro [S]	99	1974–1994	[41]	Med	96 (97.0)	24 (24.2)	10.5	PRN	24.0
Sailer 2003 (128)	Austria	Retro [S]	113	1991–2001	[[49.8]	Med	55.4 (49.0)	3 (2.7)	[4.4]	PRL	NR
**Inflammatory diseases: inflammatory bowel disease**											
Bruewer 2003 (129)	Germany	Retro [S]	73	1982–2000	[32.9]	Med	33 (45.2)	2 (2.7)	[0.4]	PRL	NR
Bruewer 2003 (129)	Germany	Retro [S]	146	1982–2000	[34]	Med	75 (51.4)	0 (0)	[0.1]	PRL	NR
Longo 2003 (130)	US	Retro [C]	158	1997–2001	59.0	Med	2 (1.3)	7 (4.4)	0.1	PRL	78.0
**Inflammatory diseases: polymyalgia rheumatica**											
Gonzalez-Gay 1999 (131)	Spain	Retro [S]	134	1987–1998	70.5	Med	85 (63.4)	12 (9.0)	NR	PRN	20.2
Gran 2001 (33)	Norway	Retro [S]	274	1987–1997	NR	Med	181 (66.1)	56 (20.4)	5.3	PRL	NR
**Inflammatory diseases: rheumatoid arthritis**											
McDougall 1994 (132)	Canada	Case control study [S]	122	1966–1993	41.7	Med	85 (69.7)	52 (42.6)	18.1	PRN	82.8
Bakker 2012 (133)	Netherlands	Prospective cohort [S]	117	NR	54.0	Med	70 (59.8)	1 (0.9)	[2.1]	PRN	24.0
Ajeganova 2014 (134)	Sweden	Clinical trial [S]	112	1995–2009	50.6	Registry	77 (68.8)	10 (8.9)	10.0	PRL	120.0
Listing 2015 (135)	Germany	Prospective cohort [S]	6155	2001–2011	[55.8]	Registry	5113 (83.1)	198 (3.2)	3.5	PRL	42.0
Chester 2016 (136)	US	Prospective cohort [S]	3496	1981–2006	56.9	Med	2662 (76.1)	1357 (38.8)	[5]	PRN	42.8 [24]
Roubille 2017 (137)	France	Retro [S]	386	2002–2013	47.5	Med	200 (51.8)	7 (1.8)	NR	PRL	33.0
Kim 2018 (138)	Korea	Retro [S]	2812	2000–2016	[51.5]	Med	2330 (82.9)	89 (3.2)	7.8	PRL	NR
Wilson 2019 (139)	UK	Retro [C]	13 770	1995–2015	56.2	Med	NR	2074 (15.1)	8.1	PRN	{9.5]
**Inflammatory diseases: Still’s disease**											
Kim 2012 (140)	Korea	Retro [S]	54	1996–2008	[37.3]	Med	39 (72.2)	5 (9.3)	2.2	PRL	NR
Ruscitti 2016 (141)	Italy	Retro [S]	100	2000–2015	45.4	Med	66 (66)	10 (10.0)	3.5	PRN	NR
**Hematologic diseases: Aplastic anemia**											
Gluckman 1992 (142)	France Belgium Switzerland	Clinical trial [S]	56	NR	NR	Med	NR	19 (33.9)	[1.7]	PRL	2.0
**Hematological diseases: Evan’s syndrome**											
Michel 2009 (143)	France	Prospective cohort [S]	68	2005–NR	56.4	Med	41 (61.3)	16 (23.5)	4.8	PRL	NR
**Respiratory tract diseases: asthma**											
Maunsell 1968 (144)	UK	Retro [S]	170	1952–1962	NR	Med	101 (59.4)	10 (5.9)	NR	PRN	60.3
Walsh 1966 (145)	UK	Retro [S]	245	1953–1965	[48.0]	Med	152 (62.0)	16 (6.5)	NR	PRL	12.0
**Respiratory tract diseases: COPD**											
Niewoehner 1999 (146)	US	Clinical trial [S]	80	1994–1996	68.1	Med	3 (3.8)	2 (2.5)	0.3	PRN	2.0

Abbreviations: [C], community care; COPD, chronic obstructive pulmonary disease; Med, medical record; No., number; NR, no report; PRL, prednisolone; PRN, prednisone; Retro, retrospective study; [S], secondary care; UK, United Kingdom; US, United States of America.

The median age of participants across the studies was 49.1 years, and women accounted for 64% of participants. Underlying conditions treated with chronic GC therapy were classified into 5 major pathophysiology groups: vasculitides, connective tissue diseases (CTDs), inflammatory diseases, nonmalignant hematological diseases, and respiratory diseases. Only one eligible study (n = 56) involved nonmalignant hematological diseases; this cohort was included in the overall pooled analysis but not presented as a separate subgroup due to insufficient sample size.

GC exposure varied substantially across studies and was standardized to prednisolone-equivalent doses (Supplemental 7) (14). Prednisolone was the commonly prescribed agent (57.5%), followed by prednisone (41.7%), while only 0.8% of studies used both. Dose reporting formats varied widely across studies, including cumulative dose, daily dose, short-term doses at fixed time points (3, 6, or 12 months), maintenance dose, final follow-up dose, and starting dose. Among these, the most consistently reported and clinically comparable measures were: (1) mean cumulative dose, (2) mean daily dose during follow-up, and (3) initial daily dose. These metrics were therefore selected for subsequent dose-response analyses. Considerable inconsistency was observed for several dose metrics such as median cumulative dose, median daily dose, short-term mean or median doses at 3–12 months, and median maintenance or final follow-up doses, which were each reported by only a small number of studies and therefore cataloged but excluded from further quantitative analysis (Supplemental 8) (14).

Across 34 studies reporting mean cumulative exposure (n = 25 673), cumulative dose ranged from 0.3 to 36.7 g. Initial doses varied from physiologic replacement to high-dose immunosuppression (0.1–1.5 or 5.6–258 mg/day). Duration of GC therapy also differed widely, with mean exposure ranging from 1.5 months to 10 years, and median exposure from 9 months to 9 years. The mean follow-up among studies contributing mortality outcomes was 5.0 ± 3.6 years (n = 77; range 1 month to 18.1 years).

## Risk of bias

Studies rated as low, moderate, or serious risk were included in quantitative synthesis, while studies judged as critical overall (5.2%; n = 7) were excluded according to the prespecified protocol (Supplementals 9 and 10) (14).

### Mortality risk across underlying conditions

**Figure 2:**
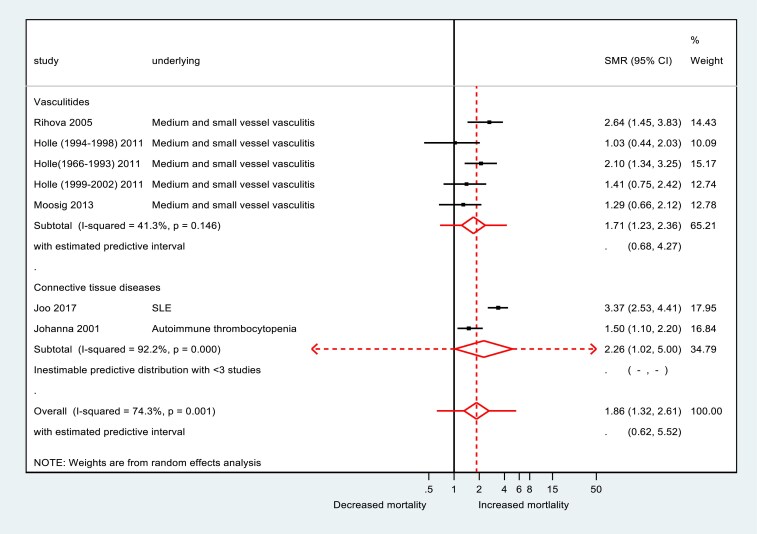
Pooled standardized mortality ratio (SMR) estimates by disease category. Forest plot showing pooled standardized mortality ratios comparing mortality in glucocorticoid-treated patients with background population mortality, stratified by disease category. Estimates were derived using random-effects meta-analysis. Squares represent study-level estimates, and diamonds represent pooled effect sizes with 95% confidence intervals. Abbreviations: CI, confidence interval; SLE, systemic lupus erythematosus; SMR, standardized mortality ratio.

Across all cohorts, chronic GC use was associated with excess mortality. The pooled SMR was 1.87 (95%CI 1.32–2.61; range 1.03–3.37; I^2^74.3%, n = 7), indicating nearly double mortality relative to background populations (Fig. 2). Significant excess mortality was also observed in disease-specific subgroups, including the vasculitides (SMR 1.71; 95%CI 1.23–2.36, *I*^2^ = 41.3%) and CTDs (SMR 2.26; 95%CI 1.02, 5.00, I^2^ 92.2%).

**Figure 3:**
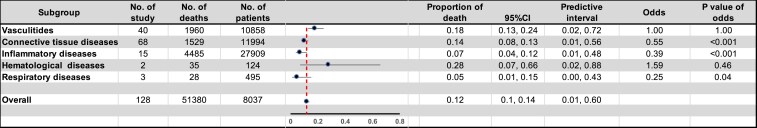
Proportion of deaths across disease subgroups. Forest plot showing pooled proportions of deaths among patients treated with systemic glucocorticoids across disease subgroups. Estimates were calculated using random-effects models with corresponding 95% confidence intervals. Abbreviations: CI, confidence interval; No., number.

The pooled proportion of deaths was 12% (95%CI 10–14%; range 0–84%; *I*^2^ = 89.1%). Mortality rates varied by disease category: vasculitides 18.0%, CTDs 14.0%, inflammatory diseases 30.0%, and respiratory diseases 5.0% (Fig. 3).

### Dose-response relationship between GC exposure and mortality

**Figure 4:**
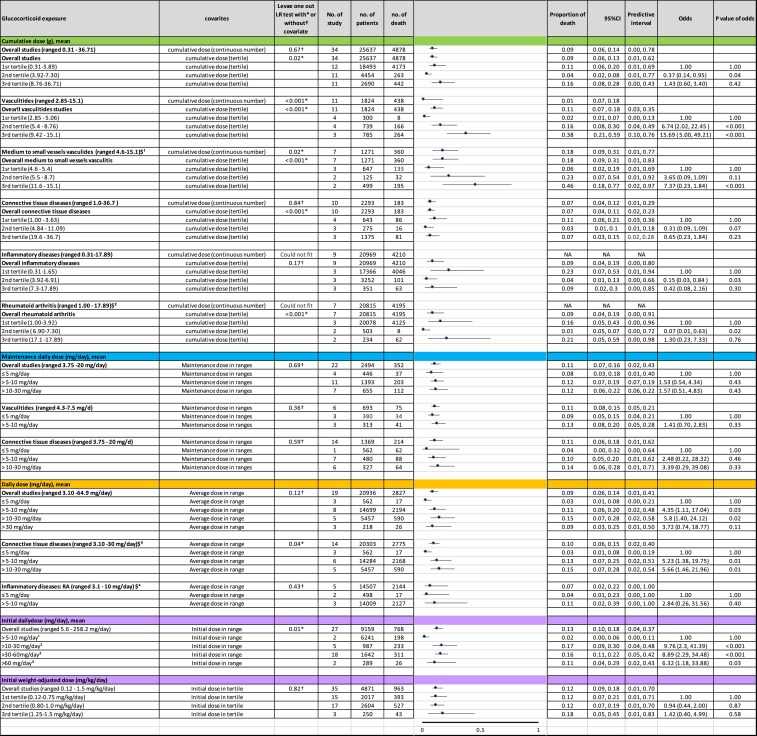
Glucocorticoid exposure metrics and subgroup-specific mortality estimates. Forest plot summarizing reported glucocorticoid exposure metrics (daily dose, cumulative dose, initial dose, and duration) and corresponding subgroup-specific mortality proportions or effect estimates. Analyses were conducted using mixed-effects meta-regression models where applicable. Prediction intervals are shown where available. Abbreviations: CI, confidence interval; CTD, connective tissue disease; GC, glucocorticoid; g, grams; HD, hematologic disease; ID, inflammatory disease; IQR, interquartile range; mg/d, milligrams per day; mg/kg/d, milligrams per kilogram per day; N, number of patients; n, number of studies; OR, odds ratio; PI, prediction interval; RD, respiratory disease; SD, standard deviation; SMR, standardized mortality ratio; VD, vasculitis.

A consistent dose-response relationship between GCs and mortality was observed across multiple GC exposure metrics, although the magnitude and clarity of association varied by how exposure was quantified. Among the metrics reported as mean values, 4 domains, including cumulative dose, average daily dose during follow-up, maintenance dose, and initial dose, were the most frequently reported and most consistently associated with mortality (Fig. 4). Together, these metrics were used by over 80 studies encompassing more than 40 000 patients, providing sufficient numerical strength and comparability for inclusion in the quantitative meta-analysis. Other dosing metrics were reported inconsistently or by very few studies and were therefore cataloged descriptively but excluded from further synthesis.

Cumulative dose demonstrated the most robust association with mortality. Median cumulative doses were broadly similar across disease groups, which were 5.5 g in vasculitides, 5.5 g in CTDs, and 5.0 g in inflammatory diseases. Regression analysis confirmed cumulative dose as a significant predictor of death (*P* = .02). When categorized into tertiles, vasculitides cohorts demonstrated a strong dose-response gradient. Compared with cumulative exposure <5.1 g, mortality risk increased sharply in the middle tertile (5.4–8.8 g; OR 6.7) and highest tertile (9.4–15.1 g; OR 15.7), with both associations highly significant (*P* < .001).

Among inflammatory diseases, higher cumulative doses predicted increased mortality in studies published before 1990, whereas this association was attenuated in studies conducted during the biologic therapy era. No consistent relationship was observed in CTDs, likely due to considerable diagnostic heterogeneity.

Average daily dose during follow-up also correlated with mortality. Nineteen studies (n = 20 936) reported this metric. Median daily doses ranged from 5.5 mg/day in inflammatory diseases to 22.9 mg/day in CTDs. Compared with ≤5 mg/day, daily doses of 5–10 and 10–30 mg/day were associated with higher odds of death (OR 4.35 and 5.8, respectively). The gradient was more pronounced in inflammatory diseases, with ORs of 5.23 and 5.66, respectively (*P* = .01), underscoring that even doses traditionally regarded as low-dose chronic therapy may confer long-term mortality risk.

Maintenance dose, reported in 22 studies (n = 2494) with doses ranging from 3.75 to 20 mg/day, did not show a consistent relationship with mortality. This likely reflects variation in how “maintenance” was defined and the timing of dose assessment rather than a true absence of biological effect.

Initial GC dose emerged as a strong predictor of subsequent mortality. Relative to an initial dose of >5–10 mg/day, initial doses of >10–30, >30–60, and >60 mg/day were associated with substantially increased odds of death (OR 9.76, 8.89, and 6.32, respectively). These findings suggest that early treatment intensity may have lasting prognostic implications, even after subsequent tapering.

Across multiple dosing metrics, cumulative exposure, average daily dose, and initial dose demonstrated the clearest associations with mortality. Maintenance dose, by contrast, appeared less informative due to inconsistent reporting. Collectively, these findings indicate that both overall burden of GC exposure and early treatment intensity contribute meaningfully to long-term mortality risk.

### Duration of GC exposure and mortality

**Figure 5:**
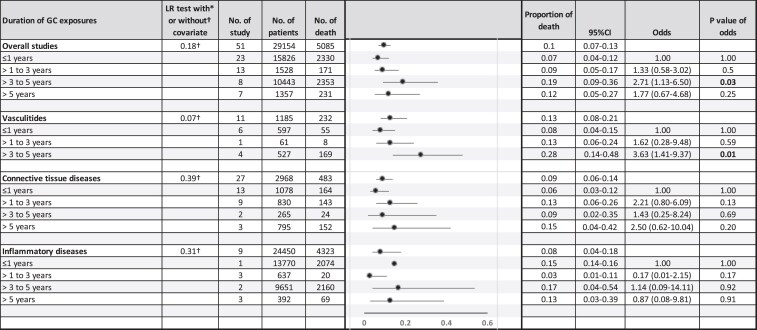
Mortality proportions stratified by duration of glucocorticoid exposure. Forest plot demonstrating pooled mortality proportions stratified by duration of glucocorticoid exposure (≤1 year, >1–3 years, >3–5 years, and >5 years). Estimates were calculated using random-effects models. Abbreviations: GC, glucocorticoid; mg/d, milligrams per day; No., number.

Duration of GC exposure refers to the length of time that patients received GC therapy. This differs from follow-up time, which reflects the total observation period, regardless of whether GC therapy was ongoing. Fifty-one studies reported mean duration of GC exposure (1.5 months to 10 years), and ten reported median duration (9 months to 9 years). Although study-level duration data limited interpretability, stratified analyses suggested an association between longer exposure and increased mortality. Exposure for 3–5 years was associated with higher odds of death (OR 2.71; 95%CI 1.13–6.50; *P* = .03) (Fig. 5). In the vasculitides, this association was stronger (OR 3.63; 95%CI 1.41–9.37; *P* = .01), whereas no significant associations were detected in CTDs or inflammatory diseases.

### Duration of study follow-up and mortality

Study follow-up duration, defined as the total observation period rather than duration of GC exposure, was not directly associated with mortality (Supplemental 11) (14). However, when follow-up was stratified, mortality increased significantly beyond the first year for all diseases combined (OR 3.45; 95%CI 1.44–8.30; *P* = .01) and for the vasculitides specifically (OR 6.26; 95%CI 2.36–16.59; *P* = .01). These findings suggest that extended follow-up enables the emergence of both disease-related and treatment-related mortality, rather than follow-up duration itself being a causal factor.

### Causes of death

**Figure 6:**
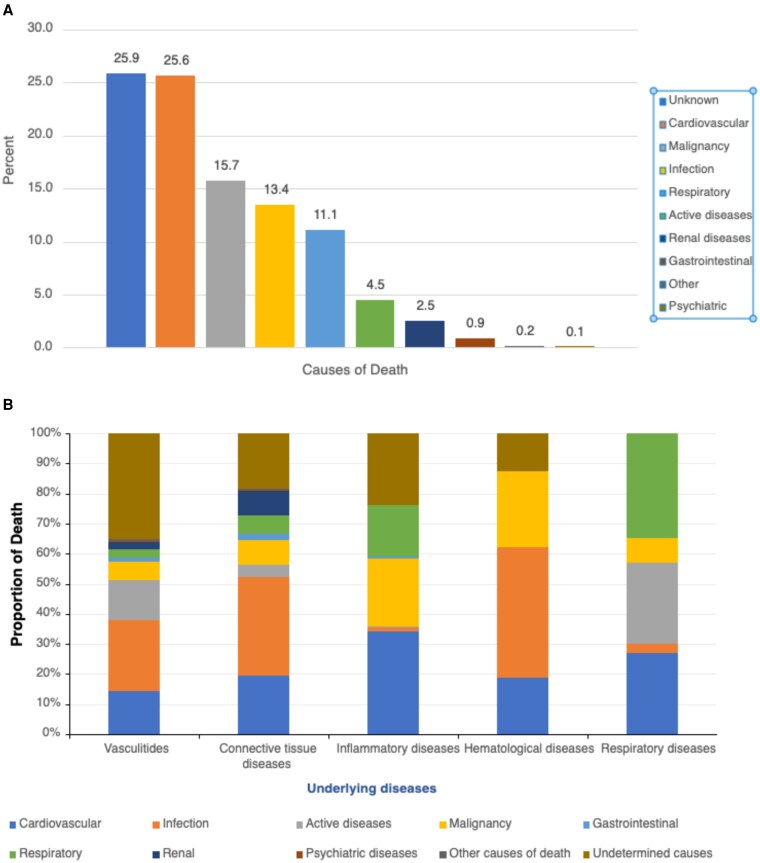
Causes of death by category and underlying disease. (A) Overall percentage distribution of causes of death across the pooled study population. (B) Proportional distribution of causes of death stratified by underlying disease category.

Eighty-three of 128 articles reported cause-specific mortality (Fig. 6A and Supplementals 12 and 13) (14). For approximately one-quarter of cases, the causes of death were unclear. Cardiovascular disease was the leading causes of death overall (25.6%), followed by malignancy (15.7%), infection (13.4%), respiratory failure (10.8%), and active underlying disease (4.5%).

Cause-of-death patterns varied substantially across disease groups (Fig. 6B). In the vasculitides, infection was the leading contributor to mortality (22.7%), followed by active disease (14.8%) and cardiovascular causes (13.5%); more than one-third of deaths (37.2%) were of unknown cause. In CTDs, infection again predominated (32.8%), with cardiovascular disease (18.0%), renal causes (9.8%), and malignancy (8.9%) contributing to the remaining deaths. Among inflammatory diseases, cardiovascular disease was the most frequent causes of death, with malignancy (22.7%) and respiratory failure (16.9%) also prominent. In respiratory diseases, mortality was driven mainly by respiratory failure (34.6%) and active disease (34.6%).

Despite these clear differences across disease categories, exploratory analyses did not reveal any consistent association between GC dose and specific causes of death. Thus, while GC exposure increased overall mortality risk, GC dose did not appear to determine the type of fatal outcome.

## Discussion

This study presents the first large-scale, comprehensive synthesis of mortality associated with exogenous GC use across immune-mediated, inflammatory, and respiratory diseases. By integrating evidence from 116 studies involving 128 cohorts and more than 51 000 GC-treated patients, we demonstrate a consistent association between chronic GC exposure and excess mortality across major disease groups for which these agents were historically routinely prescribed.

A key finding is the presence of reproducible dose-response relationship across multiple diseases. Cumulative exposure showed the clearest gradient, particularly in the vasculitides, where cumulative doses exceeding 5 g prednisolone-equivalent dose were associated with markedly increased mortality. Even chronic “low-dose” therapy, often perceived as relatively safe, was associated with measurable excess risk. Among inflammatory diseases such as RA, mean daily doses above 5 mg/day were associated with substantial mortality. These observations align with contemporary population-based work from the United Kingdom and Europe, in which cumulative prednisolone-equivalent dose and modest increases in daily dosing were independently associated with mortality and major adverse cardiovascular events (HR 1.26–2.05) (147, 148). Evidence from SLE similarly supports cumulative and high initial GC dose as contributors to long-term outcomes (149, 150). Collectively, these data suggest that GC exposure appears to be associated with dose-dependent long-term risk at the population level.

Confounding by indication is a key limitation in interpreting the observed associations. In clinical practice, higher GC doses are preferentially used during periods of severe or active disease, when baseline mortality risk is also elevated due to inflammatory burden, organ involvement, and comorbidity. Because our synthesis is based largely on study-level data, we could not adjust for patient-level disease activity or model time-varying GC exposure aligned to the timing of death. Consequently, the observed dose-mortality relationship may reflect, in part, the underlying disease process and its severity, and should be interpreted as an association rather than evidence of causality.

Interpretation of these findings requires caution. Mortality in the context of GC exposure is intrinsically complex and influenced by multiple competing factors. First, the underlying disease often has temporal primacy over medication effects (151); mortality risk is highest during early active disease, when GC doses are also typically highest. Second, long-term mortality reflects cumulative influences including age, comorbidities, co-treatment with immunosuppressive drugs, and chronic organ damage. Third, treatment paradigms have evolved substantially with the introduction of GC-sparing biologic therapies, such that older cohorts may reflect different prescribing practices and risk profiles compared with contemporary management (152). Finally, attribution of cause-specific mortality is inherently challenging, as deaths resulting from uncontrolled disease or comorbid conditions may be difficult to disentangle from GC-related toxicity. These considerations underscore the need for harmonized patient-level exposure data and standardized reporting in future studies (152).

Across disease groups, deaths most frequently resulted from cardiovascular disease, infection, malignancy, and active disease complications such as renal failure, pulmonary haemorrhage, or respiratory failure. Cardiovascular disease represented the leading causes of death overall, aligning with strong epidemiologic evidence linking GC exposure to increased risks of coronary disease, heart failure, and cerebrovascular events (HR 2 to 4) (10, 53, 139, 147, 153-161). Interestingly, cardiovascular mortality was highest in inflammatory diseases; predominantly RA, despite these cohorts receiving lower average daily GC doses than CTDs or the vasculitides. This pattern likely reflects the dual burden of long-standing inflammation and chronic cumulative steroid exposure, both of which accelerate atherosclerosis, and increase cardiovascular event risk (162-165). Inflammation linked to atherosclerosis may underpin plaque formation and prothrombotic states (166, 167). Current European Alliance of Associations for Rheumatology guidelines emphasize inflammation suppression as the primary preventive strategy in RA (168), but evidence regarding GC dose thresholds remains inconsistent (161, 168). Our findings contribute new evidence suggesting that GC-related cardiovascular risk is dose-dependent and persists across disease categories.

Importantly, our findings should not be interpreted as practice-altering evidence that lowering GC dose will reduce mortality across all indications. In several immune-mediated inflammatory diseases such as RA, inadequate suppression of inflammation may itself increase cardiovascular risk. Therefore, the appropriate clinical inference from these observational data is not indiscriminate GC dose reduction, but rather GC stewardship, defined as optimizing the lowest effective exposure while maintaining adequate disease control, together with proactive cardiovascular and infection risk mitigation in patients who require prolonged systemic GC therapy.

Infection was another major contributor to death in GC users, particularly in the vasculitides, CTDs, and nonmalignant hematological diseases, where high GC doses and concomitant immunosuppression are common. In contrast, infection-related mortality was low in inflammatory diseases, which generally received lower mean GC doses and had shorter follow-up periods (169). Unfortunately, most studies lacked data on organism type, preventive vaccination, prophylactic antibiotics, or timing of infection relative to GC exposure, representing a critical gap that warrants further investigation.

Malignancy-related mortality was most prominent in patients with inflammatory diseases (22.7%), consistent with reports in RA populations (170), whereas the association was weaker in SLE (171). Emerging evidence suggests that metabolic syndrome, a recognized consequence of chronic GC use, may synergise with chronic inflammation to amplify cancer risk (172-174). Although our analysis was not designed to evaluate metabolic pathways, these findings highlight potential biological intersections between GC therapy, metabolic dysfunction, and malignancy.

The temporal pattern of mortality further informs clinical interpretation. Mortality peaked between 3 and 5 years following GC initiation, particularly in the vasculitides. This period may represent convergence of cumulative GC toxicity, persistent inflammatory activity, and progressive organ damage. However, few studies provided longitudinal confirmation of ongoing GC exposure during this interval, limiting causal inference. Nonetheless, this timeframe may represent an opportunity for intensified cardiovascular monitoring, metabolic risk mitigation, infection prevention, and proactive GC stewardship.

Strengths of this study include its scale, methodological rigor, and application of mixed-effects meta-regression to quantify dose-response relationships across diverse immune-mediated inflammatory diseases. By incorporating single-arm pooled proportions and prediction intervals, we provide estimates that better reflect population-level uncertainty. The inclusion of multiple disease categories enhances generalizability and offers an integrated perspective on GC-associated mortality.

Several limitations warrant consideration. Most included studies were observational and at moderate-to-serious risk of bias, predominantly driven by incomplete adjustment for confounding. Important covariates such as disease activity, inflammatory burden, smoking status, body mass index, and concomitant immunosuppressive therapy were inconsistently reported. In addition, GC exposure metrics were heterogeneous, and maintenance dosing was variably defined. A major limitation is the inability to confirm whether deaths occurred during active GC exposure, as most studies lacked patient-level longitudinal dosing data. This introduces potential exposure misclassification and limits inference regarding causality.

Future studies should use designs better suited to address confounding by indication, including active-comparator new-user cohorts comparing GC-intensive strategies with GC-sparing regimens, incorporating standardized disease activity measures and time-varying exposure models to better isolate the independent contribution of GC dose to mortality risk.

## Conclusion

Chronic systemic GC exposure is consistently associated with increased mortality across multiple disease groups, with higher cumulative and early-treatment doses correlating with higher risk. However, causal attribution remains uncertain due to confounding by indication, limited disease-severity data, and exposure misclassification. These findings support GC stewardship and targeted cardiovascular and infection risk mitigation. Prospective comparative-effectiveness studies incorporating standardized disease activity and time-varying exposure modelling are needed to delineate the independent contribution of GC dose to long-term mortality risk.

## Data Availability

All data generated or analyzed during this study are included in this published article or in the data repositories listed in “References.”
